# Causal underpinnings of working memory and Stroop interference control: Testing the effects of anodal and cathodal tDCS over the left DLPFC

**DOI:** 10.3758/s13415-019-00726-y

**Published:** 2019-06-10

**Authors:** Anna Baumert, Nita Buchholz, Axel Zinkernagel, Patrick Clarke, Colin MacLeod, Roman Osinsky, Manfred Schmitt

**Affiliations:** 1 Max Planck Institute for Research on Collective Goods, Kurt-Schumacher-Str. 10, 53113 Bonn, Germany; 2 School of Education, Technical University Munich, Munich, Germany; 3 Department of Psychology, University of Koblenz-Landau, Landau, Germany; 4 School of Psychology, Curtin University, Perth, Australia; 5 School of Psychological Science, University of Western Australia, Perth, Australia; 6 Department of Psychology, University of Osnabrück, Osnabrück, Germany

**Keywords:** tDCS, Anodal stimulation, Cathodal stimulation, Left DLPFC, Stroop effect, Stroop sequence effect, Interference control, Working memory, *n*-back task

## Abstract

By means of transcranial direct current stimulation applied to the left dorsolateral prefrontal cortex, we investigated the causal role of increased or decreased excitability of this brain region for two facets of executive functions: working memory and Stroop interference control. We tested 1) whether anodal tDCS of the left DLPFC enhances working memory 15 minutes after termination of stimulation and in the absence of direct task practice under stimulation; 2) whether anodal tDCS of the left DLPFC enhances interference control, as evidenced by Stroop performance and Stroop sequence effects; and 3) whether cathodal tDCS leads to compromised executive functioning compared to anodal stimulation. In a between-subject design with 88 healthy psychology students, we compared the impact of anodal and cathodal stimulation against a sham condition, on performance on a Stroop task (during active stimulation) and on an *n*-back task (completed 15 minutes after active stimulation ended). We found significantly enhanced accuracy in the *n*-back task after anodal stimulation compared with sham, as well as speeded reactions in the Stroop tasks independent of trial type. By contrast, we found no modulation of Stroop interference effects or Stroop sequence effects. No inhibitory effects of cathodal stimulation were observed. These results support the causal role of the left DLPFC in working memory but lend no support to its involvement in Stroop interference control.

The underpinnings of executive functions in the brain, in particular of working memory and interference control, have attracted much scientific attention (Diamond, 2013; Miyake et al., 2000). The dorsolateral prefrontal cortex (DLPFC, Miller & Cummings, 2007) has been a key candidate for causal involvement in these facets of executive functions. Noninvasive methods to directly modulate neuronal excitability, such as transcranial magnetic stimulation (TMS) and transcranial direct current stimulation (tDCS), provide the means to illuminate the causal impact that particular areas of the neocortex exert on cognitive processing. In tDCS, electric currents of low density are applied by means of electrodes placed over the skull, with the goal of changing the resting membrane potential of neurons in the cortex area below the electrodes. Evidence suggests that the excitability of neurons is temporarily increased (hypopolarization) in the area under the anode but also in more widespread neural networks. Conversely, in the area under the cathode (and corresponding networks), excitability is decreased (hyperpolarization; Bindman, Lippold, & Redfearn, 1962; Nitsche & Paulus, 2001; Nitsche et al., 2003). By facilitating or inhibiting neuronal activity, causal effects of the targeted cortical regions (together with their pertinent neural networks) can be tested.

## Anodal tDCS Effects on Working Memory

More than a decade of research in cognitive effects of transcranial stimulation techniques (Medina & Cason, 2017), with a heavy focus on anodal tDCS, has led to inconsistent conclusions concerning the contribution of the DLPFC to working memory performance. Here, we focus on the potential causal role of the left DLPFC. While studies have targeted the right DLPFC and provided evidence that this area might be involved in working memory for certain contents (Wu, Tseng, Chang, Pai, Hsu, Lin, & Juan, 2014), most research has addressed the left DLPFC.

Several studies have found that anodal tDCS over the left DLPFC can increase performance in working memory tests, although sometimes this effect is observed only on certain measures under particular conditions (e.g., effects on reaction times, but not on accuracy of responses, Brunoni & Vanderhasselt, 2014; effects on accuracy only with higher current density or charge, Dedoncker, Brunoni, Baeken, & Vanderhasselt, 2016; effects after stimulation has ended, but not during stimulation, Hill, Fitzgerald, & Hoy, 2016; effects only when stimulation is paired with training on the specific task, but not without training, Mancuso, Ilieva, Hamilton, & Farah, 2016). It is difficult to systematically compare findings across studies because of different stimulation montages (e.g., different placements of the reference electrode) and differences between the cognitive tasks that have been employed.

Past research using *n-*back tasks (Jaeggi, Buschkuehl, Perrig, & Meier, 2010) implicates involvement of the left DLPFC in working memory (Rottschy et al., 2012). In these tasks, participants are presented consecutive numbers or letters and are instructed to respond when the present number or letter matches with one presented *n* steps earlier. Accuracy and latency to execute correct responses index the ability to hold information active over a short period of time and update it flexibly (Baddeley, 2000). A recent meta-analysis of such studies suggests that effects of anodal tDCS on working memory might be more reliably observed after stimulation has ended than during the period of active stimulation (Hill et al., 2016). Understanding the timing of anodal tDCS effects on working memory is relevant for theoretical and applied reasons. If effects persist after stimulation, then applied treatments involving tDCS delivery may be able to capitalize on enhancement of cognition that endures beyond the limited time span of active stimulation. However, it is unclear whether such aftereffects occur only indirectly, through tDCS enhancement of practice on a working memory task during active stimulation, or whether effects persist beyond stimulation without specific practice of working memory under stimulation. Discriminating the validity of these alternative possibilities is important if we are to understand how anodal tDCS affects cognitive functioning.

Studies have indicated that the effects of tDCS on the motor cortex can persist up to 1 hour after termination of stimulation (Nitsche & Paulus, 2001), with aftereffects depending on changes in membrane polarization and synaptic plasticity (Stagg & Nitsche, 2011). Similarly, several studies have reported aftereffects of anodal tDCS applied to the left DLPFC on working memory assessed with *n*-back tasks (Hoy et al., 2013; Keeser et al., 2011; Mulquiney, Hoy, Daskalakis, & Fitzgerald, 2011; Ohn et al., 2008). Those studies that tested aftereffects beyond immediate termination of stimulation found beneficial effects of anodal tDCS, compared with a sham^1^ condition, to persist 20 minutes and more after stimulation ended. Specifically, Ohn et al. (2008) observed enhanced *n*-back accuracy 30 minutes after stimulation ended, and Hoy et al. (2013) reported increasingly speeded *2*-back reaction times from immediately after stimulation ended, to 20 and 40 minutes after stimulation ended. These results support the hypothesis that tDCS has lasting effects, not only in motor cortex but also in the DLPFC, which is involved in higher mental processes. Tapping into such potentially delayed aftereffects, in the present study, we tested aftereffects at 15 minutes after stimulation ended.

However, due to the design of some of the previous studies, the basis of these aftereffects remains uncertain. In the studies by Hoy et al. (2013) and Ohn et al. (2008), participants repeatedly worked on *n*-back tasks or another working memory task (i.e., Sternberg task), under active tDCS stimulation and again after termination of stimulation.^2^ One potential explanation for the observed aftereffects is that active tDCS stimulation increased the benefits of practicing specific working memory tasks and through this indirect pathway served to enhance task skills demonstrated after stimulation ended. This explanation would fit with results from a meta-analysis, indicating that in healthy samples, anodal tDCS of the left DLPFC enhances the effectiveness of working memory training (Mancuso, Ilieva, Hamilton, & Farah, 2016). The present study was designed to assess whether augmenting left DLPFC activity in the absence of specific working memory practice, yet while working on a different cognitive task (a Classical Stroop interference task), contributes to poststimulation changes in working memory capacity. We opted for a between-subject design (with randomized assignment to anodal, cathodal, and sham stimulation), which allowed us to avoid repeated presentation of the same task. We tested poststimulation effects (15 minutes after stimulation ended) by means of an *n*-back task. Under stimulation, we employed a different cognitive task, namely a Stroop task, designed to require interference control. This way, any poststimulation effects could not be attributed to enhance practice in the same type of task (i.e., working memory task) during active stimulation.

## Is the Left DLPFC Causally Involved in Stroop Interference Control?

While there is evidence from stimulation studies to suggest that the left DLPFC is causally involved in working memory capacity in healthy populations (Hill et al., 2016), it remains unknown whether this brain region also is causally implicated in inhibitory executive functions (Vanderhasselt, De Raedt, & Baeken, 2009). Neuroimaging studies have suggested that, while other brain regions (such as the anterior cingulate cortex and the inferior frontal junction, Cieslik, Mueller, Eickhoff, Langner, & Eickhoff, 2015; Derrfuss, Brass, Neumann, & Cramon, 2005) are responsible for the detection and monitoring of stimulus and response conflicts, DLPFC activity is involved in attentional control, by shielding against task-irrelevant information (MacDonald, Cohen, Stenger, & Carter, 2000; Milham, Banich, & Barad, 2003; Nee, Wagner, & Jonides, 2007). A behavioral paradigm widely used to assess variation in specific facets of inhibitory functions is the Classic Stroop task (Stroop, 1935). Responding to incongruent items in the Stroop task (i.e., naming the text color when the meaning of the displayed word is a different color) requires actively maintaining goal-related information while competitively inhibiting processing and responding to word content (Friedmann & Miyake, 2004; Munakata, Herd, Chatham, Depue, Banich, & O’Reilly, 2011; Nigg, 2000; referred to here *as interference control*).

Studies employing the Classic Stroop have found that modulating left DLPFC activity in healthy adults impacts on overall reaction times but does not alter the Stroop interference effect (i.e., the slowing of responses in incongruent compared with congruent trials; Loftus, Yalcin, Baughman, Vanman, & Hagger, 2015; Vanderhasselt, De Raedt, Baeken, Leyman, & D’haenen, 2006). Fecteau et al. (2007, 2013) also found that anodal tDCS did not reduce Stroop interference but they did not report whether it served to improve overall reaction times. Also, Jeon and Han (2012) reported consistent results as in their study word naming times were enhanced due to anodal tDCS, in trials with interference, as well as in trials without interference.

Recently, it was argued that previous failures to detect reductions in the Stroop interference effect when anodal tDCS was applied to the DLPFC may reflect limitations of study design and test power (Frings, Brinkmann, Friehs, & van Lipzig, 2018). In line with this argument, Frings and colleagues reported a study in which they found that anodal compared with cathodal tDCS, over the left DLPFC, modulated the Stroop interference effect immediately after stimulation ended, as expressed by color classification errors in the incongruent condition compared with the congruent condition. They employed an adapted version of the Stroop task with less decision options and considerably more trials than in classical versions to improve reliability and internal validity of assessment. However, their study did not involve a sham condition, so it remains unclear whether such modulation reflects the beneficial impact of anodal tDCS.

Interestingly, Frings and colleagues also explored the impact of their tDCS manipulation on the so-called Stroop sequence effect. This sequence effect reflects the relative slowing of color classification responses on congruent trials (text-word match) when the preceding trial is incongruent (text-word mismatch) rather than congruent; and the relative speeding of color classification responses on incongruent trials when the preceding trial is incongruent rather than congruent (Egner, 2007). One explanation for these sequence effects implicates the temporary up-regulation and down-regulation of interference control (Botvinick, Braver, Barch, Carter, & Cohen, 2001). Hence, investigating whether such sequence effects are modulated by tDCS under stimulation offers an additional way of determining whether the left DLPFC is causally implicated in interference control, complementing research on modulation of the classic Stroop interference effect. Frings and colleagues did not find any modulation of Stroop sequence effects by tDCS, but again did not include the sham condition necessary to distinguish an impact of anodal and cathodal tDCS.

Notably, previous studies have investigated tDCS effects on interference control by applying the Stroop task after stimulation had ended. We wanted to know whether modulation of the Stroop effect and Stroop sequence effect would occur under active stimulation.

## Does Cathodal tDCS over the Left DLPFC Affect Working Memory or Interference Control?

Many tDCS studies investigating the involvement of the DLPFC in executive functions have focused on anodal stimulation. In principle, cathodal tDCS could have the opposite effect of anodal stimulation, with hyperpolarization of neuronal membranes serving to decrease neuronal excitability under the electrode and in related networks (Stagg & Nitsche, 2011). However, to date there is little evidence to support the hypothesis that cathodal tDCS over the left DLPFC impedes working memory or interference control. Several studies found no significant effect of cathodal stimulation compared to sham on working memory performance in an *n*-back task (Fregni et al., 2005; Keshvari et al., 2013; Mylius et al., 2012; Zaehle et al., 2011), with each study employing a different methodological setup (design, electrode montage, timing of *n*-back task, etc.). However, Hammer, Mohammadi, Schmicker, Saliger, and Münte (2011) did find decreased memory performance after learning in a cathodal condition compared with sham, and Zaehle et al. (2011) suggested that performance enhancement due to task repetition might be hampered by cathodal stimulation of the left DLPFC. Also, the modulation of the Stroop interference effect, observed by Frings et al. (2018), could plausibly have been driven by increased interference in the cathodal condition, rather than by reduced interference in the anodal condition. To determine the impact of anodal and cathodal tDCS, applied to the left DLPFC, on working memory and interference control, it is necessary to compare anodal and cathodal stimulation to a sham condition.

## The Present Study

Our study had three key goals. First, based on the observation that anodal tDCS effects on working memory can persist even in timely distance after the termination of active tDC stimulation (Hill et al., 2015; Hoy et al., 2013; Ohn et al., 2008), we wanted to determine whether aftereffects occur 15 minutes after stimulation in the absence of practice under stimulation in the same or a similar working memory task. The second goal was to determine whether the left DLPFC causally contributes to interference control. For this purpose, we tested whether anodal tDCS served to reduce interference in a Classical Stroop task completed during stimulation. As an additional measure of interference control, we also assessed sequences effects in an appropriately configured version of the Stroop task (Egner, 2007), and tested their modulation by tDCS. Third, we wanted to differentiate the impact of anodal from cathodal tDCS. Cathodal stimulation has been hypothesized to yield opposite results from anodal stimulation (Stagg & Nitsche, 2011). Unfortunately, some tDCS research on the role of DLPFC for working memory or interference control has focused exclusively on anodal stimulation, and those studies that also involved cathodal stimulation have been inconclusive, partly because of design limitations, such as the absence of a sham condition (Beeli et al., 2008; Fregni et al., 2005; Frings et al., 2018; Hammer et al., 2011; Kincses et al., 2004).

In the present study, we contrasted anodal and cathodal stimulation with a sham condition in a between-subjects design. Within each experimental condition, this design does not allow controlling for preexisting interindividual differences in working memory capacity and, thus, had the disadvantage of reduced power compared with a within-subjects design. Therefore, we employed an appropriately sized sample (*n* > 25 per group) that provided sufficient power for our critical tests. Critically, with a between-subjects design with randomized assignment to conditions, we avoided repeated presentation of the same task, thus disentangling stimulation effects on working memory from enhancement of task practice. To address the possibility that potential differences between stimulation and sham conditions could be due to the subjective experience of stimulation (Frings et al., 2018), we asked participants how they experienced the stimulation.

**Fig. 1:**
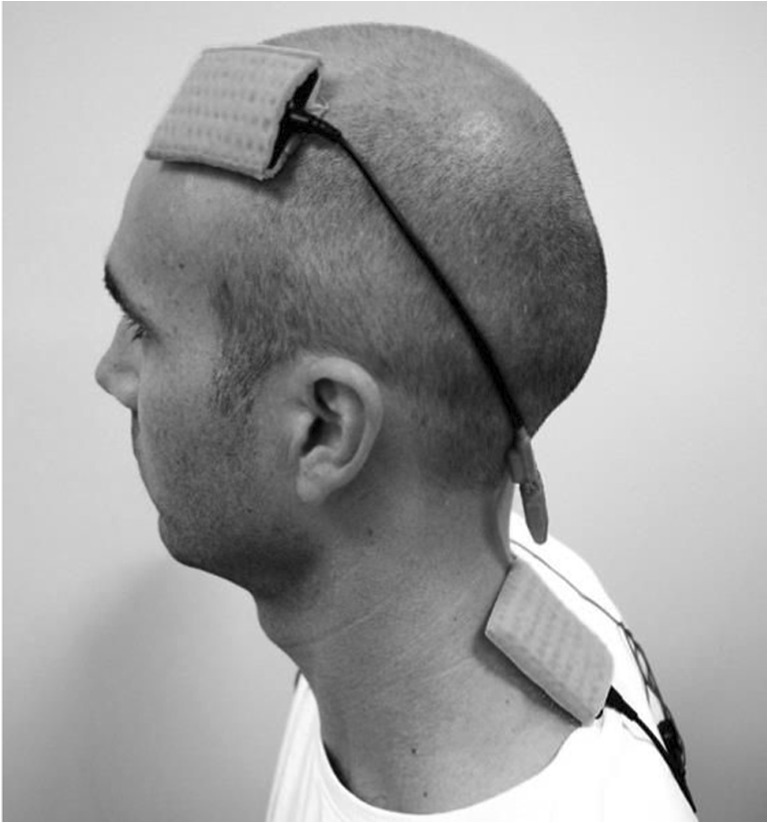
Placement of the electrodes

We followed a standard procedure for the placement of the electrode over the left DLPFC and the application of current (duration, density, electrode size; Angelakis & Liouta, 2011; Nitsche et al., 2008). We chose the placement of the reference electrode, such as to rule out the potential confound that effects could be due to opposite polarity stimulation under the reference electrode. While bilateral stimulation of the left and right DLPFC exists in the literature (Keshvari et al., 2011; Loftus et al., 2015), such an electrode montage means any effects from left DLPFC stimulation are confounded with inhibition effects of the right DLPFC, and conversely, any stimulation effects of right DLPFC are confounded with effects of left DLPFC inhibition. Therefore, we placed the reference electrode ipsilaterally between neck and shoulder (Figure 1). During stimulation, participants completed a classic Stroop task that included additional trials tailored to capture sequence effects. Fifteen minutes after the end of stimulation, we administered an *n*-back task.

Regarding aftereffects of tDCS stimulation on working memory performance, we predicted higher accuracy and speeded reactions in the *n*-back task after anodal tDCS compared to the sham condition (Hypothesis 1a and 1b). Regarding performance in the classical Stroop, we predicted that anodal tDCS (compared to the sham condition) would reduce the Stroop interference effect, as observed on measures of accuracy and response latency (Hypothesis 2a and 2b). In addition, we predicted reduced overall response latencies during anodal tDCS compared with the sham condition, without a loss in overall accuracy (Hypothesis 3).

We expected a Stroop sequence effect to occur, as revealed by an interaction between current and preceding trial type on reaction times. Specifically, we anticipated that responses to incongruent trials would be slowed disproportionately relative to responses on congruent trials when the preceding trial was congruent, rather than incongruent. Building on the research of Frings et al. (2018), we tested whether this interaction would be modulated by anodal tDCS. By enhancing interference control, anodal tDCS was expected to enlarge the Stroop sequence effect, compared to sham (Hypothesis 4).

For all tests, we also investigated whether cathodal tDCS had the opposite effects of anodal tDCS as compared to sham.

## Method

All material, data, and script is available online at https://osf.io/ta5x4/.

## Participants and Design

Ninety-six healthy psychology students (73% female) participated in return for course credit. Ages ranged from 18 to 53 years (*M* = 23.04, *SD* = 5.96). In accordance with tDCS ethics approval, individuals who were pregnant, had metal implants in their head, or suffered from neurological, internal, or psychiatric diseases were excluded from participation. Written, informed consent was obtained from all participants before the start of the study. The study protocol was approved by the local Ethics Committee.

The study involved a between-subjects experimental design with three conditions: sham; anodal stimulation; cathodal stimulation. Random assignment resulted in 31, 31, and 34 participants, respectively. Data from eight participants were subsequently omitted from analyses. Of these eight, three participants reported difficulties in understanding the cognitive tasks. For three participants, there were technical problems with stimulation or data recording. Two more participants were excluded because of color-blindness.

For an expected effect size of *f* = 0.35 and a critical alpha-level of 0.05, tests for differences between experimental conditions by means of univariate ANOVAs had a power of 1-β = 0.83; and tests for interactions between experimental condition and trial type as within-subject factor had a power of 1-β = 0.99 (G*Power 3.1.9.2; Faul, Erdfelder, Lang, & Buchner, 2007).

## Apparatus

Current was generated by a battery driven, constant current stimulator (DC-Stimulator neuroCon, Ilmenau, Germany) and induced via a pair of saline soaked sponge electrodes (35 cm^2^). One electrode was fixed over the left DLPFC (F3 according to the 10-20 EEG system). The reference electrode was placed on the ipsilateral upper trapezius muscle, between neck and shoulder (Figure 1). In the anodal stimulation condition and cathodal stimulation condition, a constant current of 1 mA (current density 0.029 mA/cm^2^) was applied for the duration of 20 minutes, including 10 s fade-in and 10 s fade-out. In the sham condition, subjects received fade-in, stimulation, and fade-out, for 30 seconds each.

Note that some tDCS studies have used higher current density than we did (Dedoncker et al., 2016, reported a range of 0.02 to 0.08 mA/cm^2^ across studies). However, our protocol was informed by the finding that, in the motor cortex, cathodal stimulation of 1 mA current applied during 20 minutes resulted in reduced neuronal excitability while higher stimulation did not (Batsikadze, Moliadze, Paulus, Kuo, & Nitsche, 2013). Also, Hoy et al. (2013) reported larger effects of anodal stimulation of the left DLPFC on working memory performance for 1 mA than for 2 mA, indicating a potential curvilinear effect of stimulation dose. Moreover, ethical considerations caution against higher densities, because they could be painful (Angelakis & Liouta, 2011).

## Measures

### Stroop tasks

The Stroop task consisted of two parts, which were not readily distinguishable for participants.

### Classical Stroop task

The first 145 trials belonged to a classical Stroop tasks. Single German color-words “rot” (red),” grün” (green), “gelb” (yellow), “blau” (blue), or nonwords were presented in randomized sequence on a black computer screen. Participants were instructed to determine the color that an item was written in by pressing as fast as possible predetermined keys on the keyboard. They used the four fingers of their dominant (for all our particiants, their right) hand to press the adjacent keys *V* for green (index finger), *B* for blue (middle finger), *N* for red (ring finger), and *M* for yellow (pinky). Items remained on the screen until one of the four keys was pressed. If the response was correct, the next trial started immediately. If the response was wrong, participants received an error message and waited three seconds for the next trial to start.

The combination of font color and word type resulted in three kinds of trials: a) 48 *congruent trials* with font color identical to meaning of the word (e.g. the word “red” written in red); b) 48 *incongruent trials* in which the meaning of the word was inconsistent with its font color (e.g., the word “red” written in blue); and c) 48 *control trials* consisting of colored nonwords.^3^ The first trial of the task served as practice.

### Stroop Sequence Effects

The second part of the Stroop task consisted of 96 congruent and incongruent trials (no control trials) which were arranged in a predetermined fixed order to permit analyses of sequence effects. Four trial types were created by this fixed order: a) congruent trials subsequent to a congruent trial (cc); b) congruent trials subsequent to an incongruent trial (ic); c) incongruent trials subsequent to a congruent trial (ci); and d) incongruent trials subsequent to an incongruent trial (ii). Across the 96 trials, each of these 4 trial types was delivered 24 times.

For data analyses, across both parts of the Stroop task, we excluded as outliers reactions which fell 3 standard deviations (SD) or more above or below the individual mean reaction time across all Stroop trials (i.e., across classic and sequence trials, *n* = 285 trials). Response latencies were computed only from trials on which participants made the correct response. Separately for both parts, we computed mean latency to respond correctly on each trial type, and we expressed accuracy as percentages of correct responses on each trial type.

### *N*-back task

We adopted the procedure from Jaeggi et al. (2010)**.** Participants were presented single consonants in white font on a black screen. Each letter was presented for 3,000 ms, with a 2,500-ms interstimulus interval, before the next letter appeared automatically on the screen. Participants were instructed to pay attention to whether a presented consonant was identical to the stimulus *n* positions prior in the sequence. They were asked to react as fast as possible by pressing the space bar on the keyboard in cases of identical stimuli but to strike no key in cases of nonidentical stimuli.

The task consisted of nine blocks, with three consecutive blocks for each level of *n*, 1-back, 2-back and 3-back, in ascending order. A block comprised 16 to 18 nontarget trials (displaying a nonidentical consonant) and 5 target trials. Across all blocks, we calculated individual mean response latencies for correct responses (i.e., key strikes in case of a match between the consonant presented and *n* positions back). Also across all blocks, accuracy was calculated as proportion of correct responses. We counted correct reactions to target trials and correct nonreactions to nontarget trials and divided by the total number of trials. Note that this common procedure in *n*-back tasks results in high mean levels of accuracies, due to the fact nonresponses are correct in the majority of trials (see Jaeggi et al., 2010).

### Questions on subjective experience

To probe for differences in subjective experience when working on Stroop and *n*-back, participants were asked to indicate how difficult and effortful they found each task and how well they had managed to concentrate during each task, with response options from 1 (*not at all*) to 6 (*very much*). Furthermore, participants were asked as how arousing (1 / *not arousing* to 6 / *very arousing*) and how pleasant or unpleasant (1 / *very unpleasant* to 6 / *very pleasant*) they found the tDCS stimulation. Finally, they indicated for each musical pieces they listened to during waiting (see below) how arousing and pleasant (−3 / *not arousing, very unpleasant* to +3 / *very arousing, very pleasant*) they had experienced it, with one respective item per musical piece.

### Procedure

Participants were tested individually in the laboratory. After providing informed consent, they sat in front of a computer screen. The experimenter attached the two electrodes to the head and shoulder of the subject and started the experiment. First, participants read the instructions for the Stroop task and completed a short practice. Then, the tDCS stimulation or sham procedure was delivered. The test block of the Stroop task started 8 minutes later. This was done to ensure that, in the anodal and cathodal conditions, the stimulation ended simultaneously with the completion of the Stroop tasks, except for small variations depending on the paticipants’ speed of responding. During this eight minute waiting period, participants listened to music (Beethoven’s Symphony No. 7 in A major, Op. 92), intended to prevent cognitive effort during waiting.

After completion of the Stroop tasks, there was a 15-minute interval, during which participants again listened to music (Beethoven´s Symphony No. 3, op. 55) to reduce the tedium of waiting, before the *n*-back task started automatically on the computer screen. After completion of the *n*-back task, participants answered questions on their subjective experience of the tasks and music and provided demographic information. Finally, they were fully debriefed and thanked.

## Data Analyses

Analyses were conducted with SPSS (Version 25) and *R* (Version 3.2.2, 2015) and the corresponding R packages *car*, *lsr, plyr*, and *nlme*. For our dependent measures in *n*-back and Stroop tasks, we screened for outliers by inspecting boxplots and excluded outliers which deviated more than 3 SD from the mean of the condition.

Furthermore, we inspected the distributions of our dependent measures in *n*-back and Stroop tasks (Table 1). Skew, Kurtosis, and Kolmogorov-Smirnov tests indicated that deviation from normality was nonsignificant for response latencies and modest for accuracy in the *n*-back task, but deviation from normality was substantial for accuracy in Stroop trials. Conducting ANOVAs for testing our hypotheses rests on the assumption that sampling distributions of means are normally distributed. For sample sizes *n* > 25 per group, this assumption can be retained even when there are modest deviations from normality in the distribution of raw scores of a variable (Tabachnik & Fidell, 2012). Therefore, we proceeded with ANOVAs in cases of significant deviation from normality. For accuracy in Stroop trials, we additionally checked the robustness of our findings, by means of a nonparametric test of median differences.

**Table 1:** Indicators of shape of distribution for main dependent variables

Variable	*M*	*Median*	*Skew*	*Kurtosis*	*K-S test statistic*	*p*
*n*-back accuracy	0.92	0.93	-0.31	-0.74	0.12	0.005
*n*-back RL	586.33	573.62	0.90	1.15	0.10	0.046
Stroop accuracy congruent trials	0.97	0.98	-1.31	2.28	0.23	<0.001
Stroop accuracy incongruent trials	0.96	0.98	-1.57	2.72	0.21	<0.001
Stroop accuracy control trials	0.97	0.98	-1.10	0.58	0.25	<0.001
Stroop RL congruent trials	604.58	604.65	0.28	-0.09	0.07	0.20
Stroop RL incongruent trials	657.85	661.41	0.26	-0.29	0.05	0.20
Stroop RL control trials	626.53	631.58	0.21	-0.05	0.06	0.20
Sequence Stroop RL cc	607.92	614.04	0.20	-0.44	0.09	0.10
Sequence Stroop RL ic	612.25	611.91	0.19	-0.56	0.06	0.20
Sequence Stroop RL ci	673.84	671.45	0.37	0.03	0.06	0.20
Sequence Stroop RL ii	666.71	667.83	0.26	-0.32	0.07	0.20

K-S test = Kolmorov-Smirnov test of normality of score distributions

For *n*-back task accuracy and latency data, we conducted one-way ANOVAs with tDCS condition (sham/anodal/cathodal) as the between-subject factor. For Stroop task accuracy and latency data, we conducted two-way ANOVAs with tDCS condition (sham/anodal/cathodal) as the between-subject factor and trial type (congruent/incongruent/control) as the within-subject factor. Finally, we analyzed the response latency data revealing Stroop sequence effects using a three-way ANOVA with tDCS condition (sham/anodal/cathodal) as the between-subject factor and with preceding trial type (incongruent/congruent) and current trial type (incongruent/congruent) as the within-subject factors. For all tests, we probed for homogeneity of variances between conditions. In cases of heterogeneous variances, we report results with Welch-corrected degrees of freedom. We explored the nature of significant effects obtained in these ANOVAs by means of post-hoc *t* tests or, in case of heterogeneous variances, by means of Welch tests.

## Results

### Questions on Subjective Experience

For each question about subjective experience, a one-way ANOVA with tDCS condition (sham/anodal/cathodal) as the between-subject factor was conducted. Results are displayed in Table 2. Regarding the *n*-back task, there were no significant differences between conditions in subjective experiences. Regarding the Stroop task, participants in the cathodal condition reported that they found the Stroop task less difficult compared with participants with anodal stimulation, *t*(44.64) = −3.60, *p* = 0.001, *d* = −0.96, but not compared with participants in the sham condition, *t*(46.75) = −1.70, *p* = 0.096, *d* = −0.44. The participants in the anodal and sham conditions did not differ significantly, *t*(55) = 1.43, *p* = 0.16, *d* = 0.38. Participants in the cathodal condition reported significantly higher concentration during the Stroop task compared with the anodal condition, *t*(56) = 3.44, *p* = 0.001, *d* = 0.90, and compared with the sham condition, *t*(59) = 2.94, *p* = 0.005, *d* = 0.75. Again, those in the anodal and sham condition did not significantly differ, *t*(55) = 0.22, *p* = 0.83, *d* = 0.06.

**Table 2:** Results of univariate ANOVAs for questions on subjective experience, and means and standard deviations separate for conditions

Condition	Sham		Anodal		Cathodal		*F*(2,85)	*p*
Variable	*M*	*SD*	*M*	*SD*	*M*	*SD*		
Difficulty Stroop	2.53	1.31	3.00	1.14	2.06	0.77	5.31	0.007
Effort Stroop	4.90	0.89	4.52	0.89	4.97	0.88	2.12	0.13
Concentration Stroop	4.13	1.11	4.07	0.96	4.84	0.74	6.11	0.003
Difficulty *n*-back	4.40	1.07	4.26	1.06	4.65	0.95	1.06	0.35
Effort *n*-back	4.80	1.00	4.93	0.96	5.16	0.78	1.23	0.30
Concentration *n*-back	3.87	1.38	3.85	1.10	4.35	0.95	1.84	0.17
Arousal tDCS	2.27	1.29	2.89	1.05	3.16	1.51	3.74	0.028
Valence tDCS	3.43	1.14	3.56	1.01	4.06	1.15	2.79	0.067
Arousal music	0.38	1.64	0.41	1.24	0.31	1.31	0.04	0.96
Valence music	0.97	1.71	0.89	1.63	0.92	1.32	0.02	0.98

*n*(sham) = 30, *n*(anodal) = 27, *n*(cathodal) = 31. Response options from 1 (*not at all / very unpleasant*) to 6 (*very much / very pleasant*), except for arousal music and valence music with response options from −3 (*not arousing / very unpleasant*) to +3 (*very arousing / very pleasant*)

With regard to the subjective experience of stimulation, participants in the cathodal condition reported more arousal due to stimulation compared with the sham condition, *t*(59) = 2.49, *p* = 0.016, *d* = 0.63, but not compared with the anodal condition, *t*(56) = 0.79, *p* = 0.27, *d* = 0.21. Participants in the anodal condition also reported higher arousal than in the sham condition, *t*(54.52) = 2.01, *p* = 0.049, *d* = 0.53.

The musical pieces were experienced as mildly positive and mildly arousing, overall. There were no significant differences between conditions.

## *N*-back Task

Means and standard deviations of correct response rates and reaction latencies for each condition in the *n*-back task are displayed in Table 3.

**Table 3:** Means and standard deviations of accuracy and response latencies (RL in ms) in *n*-back task

Condition	Sham		Anodal		Cathodal	
Variable	*M*	*SD*	*M*	*SD*	*M*	*SD*
*n*-back accuracy	.90	.04	.93	.03	.92	.05
*n*-back RL	592.37	117.45	563.49	104.84	585.97	120.25

*n*(sham) = 30, *n*(anodal) = 26, *n*(cathodal) = 31. Results after exclusion of one outlier in the anodal condition

### Accuracy

**Fig. 2:**
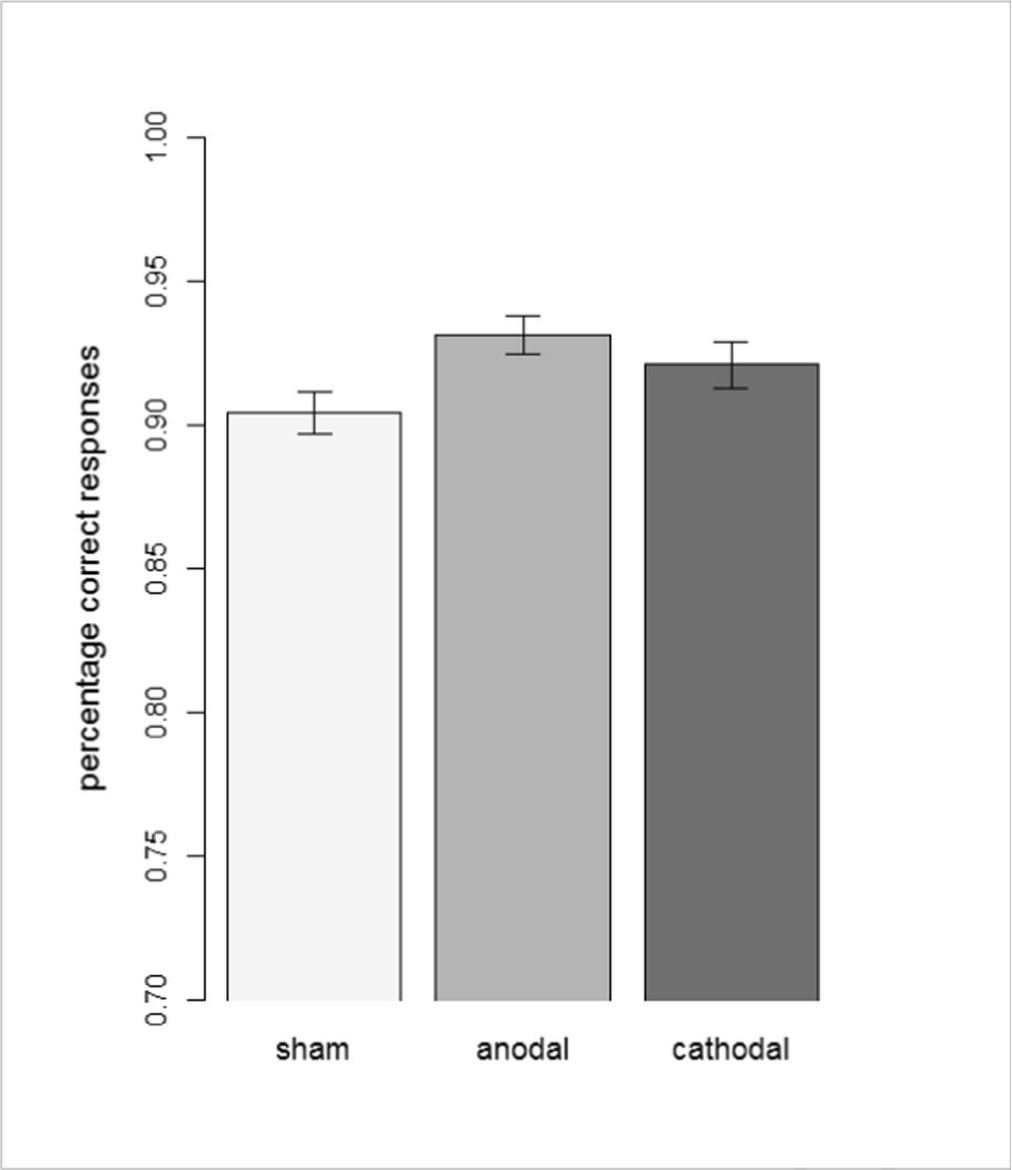
*n*-back accuracy (Percentage of Correct Responses) Separate for tDCS Conditions. (Note. Y-axis starts at score reachable by constant nonresponses.)

One outlier was identified and excluded (in the anodal stimulation condition). Before exclusion of the outlier, the main effect of stimulation condition was not significant, *F(*2,56.386) = 2.59, *p* = 0.08, η^2^ = 0.08. After exclusion, the main effect of condition was significant, *F(*2,55.898) = 4.01, *p* = 0.024, partial η^2^ = 0.13 (even when accounting for repeated testing with a Bonferroni-adjusted critical alpha-level 0.025). In line with Hypothesis 1a, a post-hoc *t* test revealed that *n*-back accuracy rate was higher in the anodal tDCS condition compared with the sham condition, *t*(54) = 2.81, *p* = 0.007, *d* = 0.85. There was no significant difference in accuracy between the sham condition and the cathodal tDCS condition, *t*(59) = 1.52, *p* = 0.13, *d* = 0.44, nor between the anodal tDCS and cathodal tDCS condition, *t*(55.95) = 0.55, *p* = 0.59, *d* = 0.26 (see Figure 2).

### Response latencies

Again, one outlier was identified and eliminated (in the anodal condition). No significant effects emerged from this analysis. Contrary to predictions of Hypothesis 1b, for correctly answered *n*-back target trials, response latencies did not differ significantly between tDCS conditions, *F*(2,83) = 0.47, *p* = 0.62, partial η^2^ = 0.01 (Table 2).

## Classical Stroop Task

Means and standard deviations for correct response rates and reaction latencies in congruent, incongruent, and control trials in each condition are displayed in Table 4.

**Table 4:** Means and standard deviations of accuracy and response latencies (RL in ms) in Classical Stroop Task

Condition	Sham		Anodal		Cathodal	
Variable	*M*	*SD*	*M*	*SD*	*M*	*SD*
Stroop accuracy congruent trials	.97	.02	.96	.04	.97	.03
Stroop accuracy incongruent trials	.96	.03	.96	.04	.97	.04
Stroop accuracy control trials	.97	.03	.97	.03	.97	.03
Stroop RL congruent trials	625.03	63.82	573.51	81.70	611.86	83.66
Stroop RL incongruent trials	684.57	66.03	624.57	91.22	660.99	104.72
Stroop RL control trials	650.92	57.26	598.48	85.10	627.36	88.02

*n*(sham) = 30, *n*(anodal) = 27, *n*(cathodal) = 31. Response latencies aggregated across correct responses. Accuracy was determined as percentage of correct responses for each trial type separately. For accuracy, three outliers were excluded (two in the anodal condition, one in the cathodal condition)

### Accuracy

Three outliers on incongruent trials were identified and excluded (1 in the anodal condition, 2 in the cathodal condition). There were no significant effects of trial type, *F*(2,81) = 0.42, *p* = 0.66, partial η^2^ = 0.01, condition, *F*(2,82) = 1.50, *p* = 0.23, partial η^2^ = 0.035, or their interaction, *F*(4,164) = 1.50, *p* = 0.20, partial η^2^ = 0.035. Other than expected, for accuracy, we did not find the anticipated Stroop interference effect. Inconsistent with Hypothesis 2a, there was no modulation of Stroop accuracy by tDCS condition. Nonparametric tests comparing medians across tDCS conditions also resulted in no significant differences for any trial type, all *p*s > 0.24.

### Response latencies

**Fig. 3:**
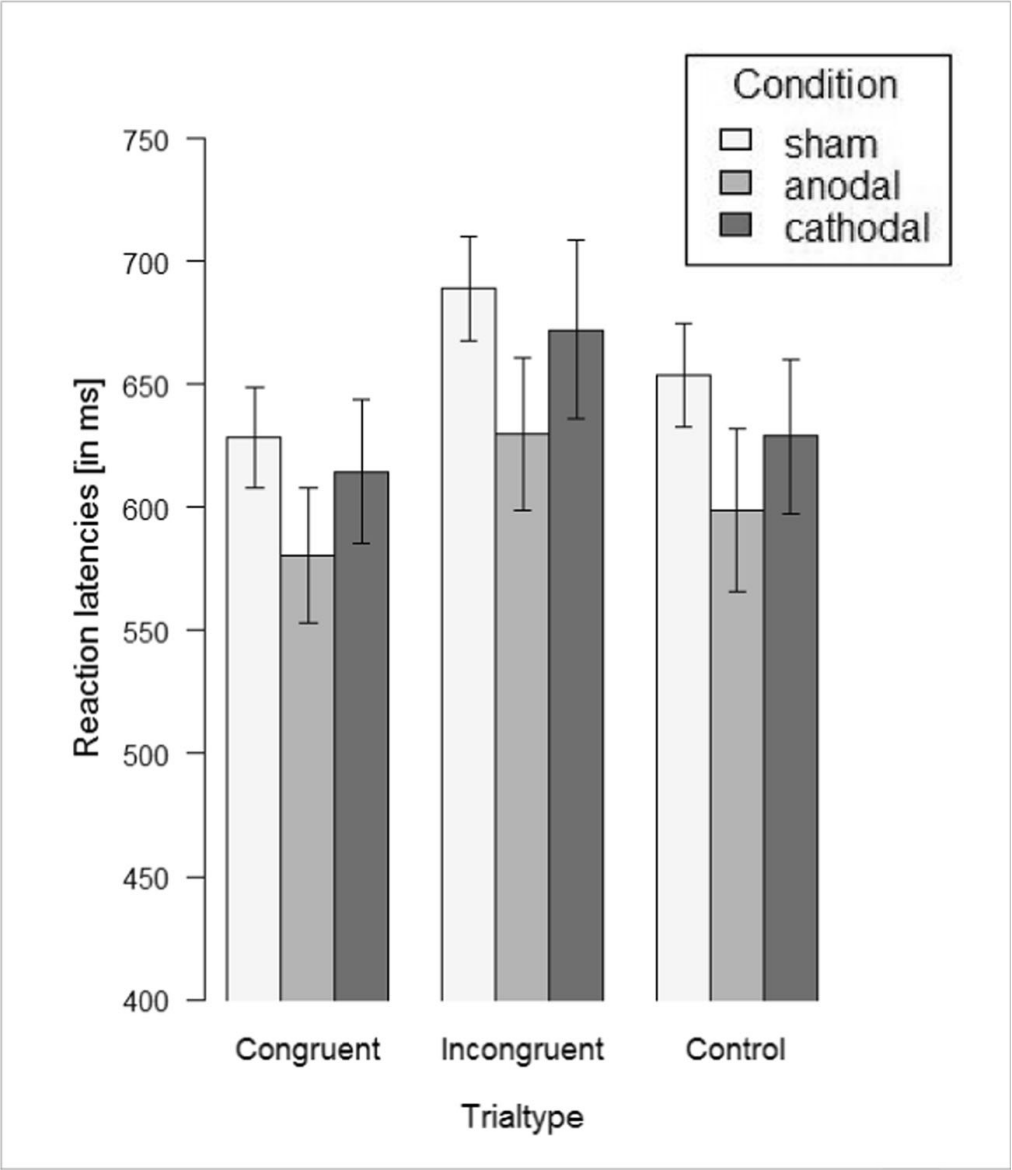
Stroop reaction latencies for congurent, incongruent, and control trials, separate for tDCS conditions

No outliers were identified. There was a significant main effect of trial type, *F*(2,84) = 99.37, *p* < 0.001, partial η^2^ = 0.54, indicating the presence of the anticipated Stroop interference effect (Figure 3). Mean color classification latencies were slower on incongruent trials than on congruent trials, *t*(87) = 12.79, *p* < 0.001, *d* = 1.51. Color classification latencies were slower on incongruent trials than on control trials, *t*(87) = 7.18, *p* < 0.001, *d* = 0.84, and were faster on congruent trials than on control trials, *t*(87) = −8.73, *p* < 0.001, *d* = −0.97.

In addition, there was a significant main effect of tDCS condition, *F*(2,84) = 3.48, *p* = 0.035, partial η^2^ = 0.076. Consistent with Hypothesis 3, the nature of this main effect was that response latencies were shorter in the anodal condition than in the sham condition (congruent trials: *t*(55) = 2.67, *p* = 0.01, *d* = 0.71; incongruent trials: *t*(55) = 2.87, *p* = 0.006, *d* = 0.76; control trials: *t*(44.83) = 2.70, *p* = 0.01, *d* = 0.73). Reaction latencies did not differ significantly between cathodal and sham condition, all *p*s > 0.22; nor between anodal and cathodal condition, all *p*s > 0.08. Importantly, the interaction of tDCS condition and trial type was not significant, *F*(4,82) = 0.65, *p* = 0.63, partial η^2^ = 0.015. Thus, contrary to Hypothesis 2b, the magnitude of the Stroop interference effect was not affected by tDCS condition.

We further explored potential effects of stimulation on Stroop interference by transforming response latencies into percentage interference and facilitation, respectively, as dependent variables. For this purpose, we took response latencies on control trials as individual baseline and subtracted it from response latencies on incongruent trials (as a measure of interference), and on congruent trials (as a measure of facilitation), respectively. Both difference scores were divided by response latencies on control trials to give the percentage interference or facilitation relative to the individual baseline. An ANOVA with the two factors condition and trial type (interference percentage / facilitation percentage) did not yield a significant effect of condition, *F*(2,85) = 0.66, *p* = 0.52, partial η^2^ = 0.015, or condition x trial type, *F*(2,85) = 0.56, *p* = 0.57, partial η^2^ = 0.01.

## Stroop Sequence Effects

Color classification latencies observed in the Stroop task component structured to reveal sequence effects are shown in Table 5. We analyzed these data by means of a three-factorial ANOVA with tDCS condition (sham/anodal/cathodal) as the between-subject factor and preceding trial type (incongruent/congruent) and current trial type (incongruent/congruent) as the within-subject factors.

**Table 5:** Means and standard deviations of response latencies (RL in ms) in sequence Stroop Task

Variable	Sham		Anodal		Cathodal	
	*M*	*SD*	*M*	*SD*	*M*	*SD*
Sequence Stroop RL cc	624.02	60.47	579.75	70.79	614.08	88.14
Sequence Stroop RL ic	622.53	61.26	591.16	65.51	617.84	81.92
Sequence Stroop RL ci	695.49	64.39	634.48	81.75	685.57	113.05
Sequence Stroop RL ii	681.14	60.64	633.39	71.27	680.73	103.81

*n*(sham) = 30; *n*(anodal) = 27; *n*(cathodal) = 31; c = congruent trial; i = incongruent trial

**Fig. 4:**
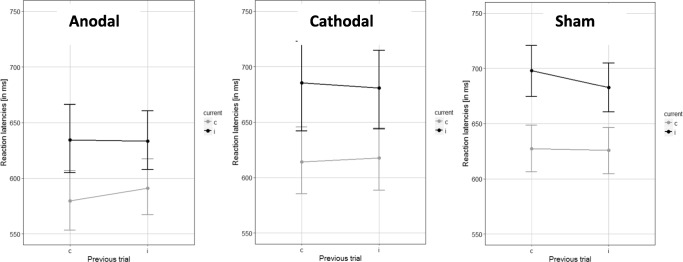
Stroop sequence effect, displayed as response latencies, separate for current trial type, previous trial type, and tDCS condition

This revealed a significant main effect of current trial type, *F*(1,85) = 172.19, *p* < 0.001, partial η^2^ = 0.67, again reflecting the classical Stroop interferences effect, with color classification latencies on congruent trials being significantly faster than those on incongruent trials. In addition, there was a significant interaction between current and preceding trial type, *F*(1,85) = 4.92, *p* = 0.029, partial η^2^ = 0.055, consistent with the expected sequence effect (Figure 4). Specifically, the interference effect reflecting slowed color classification on the incongruent compared to the congruent (current) trials was significantly bigger when these trials were preceded by congruent trials (*M* = 66.34, *SD* = 53.24) rather than by incongruent trials (*M* = 55.09, *SD* = 45.07), *t*(87) = 2.23, *p* = 0.028, *d* = 0.22.

Once again, there was a significant main effect of tDCS condition, *F*(2,85) = 3.20, *p* = 0.046, partial η^2^ = 0.07. This reflected faster color classification latencies in the anodal tDCS condition compared with the sham condition, *t*(55) = 2.86, *p* = 0.006, *d* = 0.77, consistent with Hypothesis 3. There were no significant differences in color classification latencies between the cathodal tDCS condition and sham condition, *t*(59) = 0.32, *p* = 0.75, *d* = 0.08. Importantly, there was no significant interaction effects involving tDCS condition, all *p*s > 0.19. As such, inconsistent with Hypothesis 4, there was no evidence that the magnitude of the observed sequence effect was modulated by either anodal or cathodal tDCS.

## Discussion

In the present study, we examined the impact of tDCS over the left DLPFC on the executive functions of working memory and interference control. There was no effect of tDCS manipulation of response latencies on the *n*-back task, different than predicted by Hypothesis 1b. However, our accuracy measure of working memory capacity, provided by *n*-back task performance was improved in the aftermath of anodal tDCS delivered to the left DLPFC, compared with the sham condition. This can be interpreted as support for Hypothesis 1a, while taking into account the fact that exclusion of a predefined outlier was necessary to reveal the effect. The finding indicates that anodal tDCS can have an enhancing effect on working memory performance 15 minutes after stimulation has ended (Brunoni & Vanderhasselt, 2014; Dedoncker et al., 2016; Hill et al., 2016; Mancuso et al., 2016). Importantly, because our design precluded the opportunity to practice the *n*-back task during active tDCS, the observed aftereffect of anodal tDCS cannot be attributed to enhanced training on the *n*-back task under active stimulation.

Under stimulation, participants in our study worked on Stroop trials, designed to assess interference control as an executive function distinct from working memory. Accordingly, observing enhanced working memory performance after anodal stimulation of the left DLPFC had ended could be attributed to heightened cognitive training under stimulation, which might have generalized from one specific task (Stroop) to the other (*n*-back). In other words, anodal tDCS might have improved training of executive functions more generally due to completing the Stroop task, which then persisted and transferred to more accurate responses in the *n*-back task 15 minutes after stimulation ended. However, this interpretation seems less plausible in the light of studies on training working memory, which have indicated that transfer between tasks tapping into different facets of executive functions are not robustly observed (Melby-Lervåg, Redick, & Hulme, 2016) or very small in effect size (Soveri, Antfolk, Karlsson, Salo, & Laine, 2017).

Rather, it seems plausible that anodal tDCS directly enhanced working memory capacity, with this enhancement remaining evident 15 minutes after active stimulation (Ohn et al., 2008). Alternatively, it is possible that the Stroop task served to train working memory, specifically, even though the task was not intended as a measure for working memory capacity. Arguably, fast and correct responses in the Stroop task require holding response keys active in memory (Frings et al., 2018). The pattern of results that we obtained with regard to effects of anodal tDCS on Stroop response latencies is consistent with enhanced working memory training on the Stroop task due to anodal tDCS. We will discuss these results next. In order to further disentangle the processes through which stimulation of the left DLPFC causes better working memory performance in timely distance after active stimulation has ended, a future study could use our design but without administering a Stroop task under active stimulation (Andrews et al., 2011; Jeon & Han, 2012; Keeser, 2011).

With regard to our second research question, our study revealed no evidence that anodal tDCS applied to the left DLPFC served to modulate the classical Stroop interference effect (Hypothesis 2a and 2b) or Stroop sequence effects (Hypothesis 4). The absence of such modulation does not support the idea that the left DLPFC is causally involved in interference control. If this were the case, then anodal tDCS to this region would be expected to enhance interference control under active stimulation, consequently facilitating the speed and accuracy of color naming classification on incongruent trials to a greater degree than on congruent trials. While we found no such interaction, we found speeding of color classification responses in the anodal tDCS condition compared with the sham condition, without any accuracy tradeoff, consistent with Hypothesis 3. This result is in line with the findings obtained in several previous studies that have analyzed response latencies in Stroop tasks (Jeon & Han, 2012; Loftus et al., 2015; Vanderhasselt et al., 2006).

This speeding when correctly classifying the color of presented words is consistent with the causal involvement of the left DLPFC in determination of working memory capacity. As discussed earlier in the paper, enhanced working memory capacity should facilitate fast and accurate responding in the Stroop task, independent of trial type, because the correct response keys have to be kept active in memory (Frings et al., 2018). Because we placed the reference electrode ipsilaterally and away from other cortical areas potentially involved in executive functions, our findings support the unique role of neuronal excitability in the left DLPFC.

Different from previous studies probing for modulation of Stroop interference by anodal stimulation of the left DLPFC, we tested tDCS effects under active stimulation, rather than immediately after stimulation ended. Consistent with our results, most previous studies did not find evidence that responses in trials with inconsistent word content and color were speeded more than responses in congruent trials (Fecteau et al., 2007, 2013; Loftus et al., 2015; Vanderhasselt et al., 2006). Therefore, the timing of measurement (during vs after stimulation) does not seem responsible for the absence of Stroop interference modulation by anodal tDC stimulation of the left DLPFC.

Addressing our third research question, we observed no difference between cathodal tDCS and the sham condition, either on measures of working memory capacity or measures of Stroop task performance. Together with previous studies reporting no effects of cathodal tDCS on cognitive tasks (Jacobson et al., 2012), our results could suggest that inhibitory effects of tDCS on the left DLPFC might be readily compensated through other brain regions. Banich and colleagues proposed that, in a temporal cascade, different regions in the frontal cortex exert interference control, depending on how effectively control was exerted previously through other brain regions (Banich, 2009). Concretely, this could mean that inhibition of left DLPFC activity is compensated by upregulation of activity in the anterior cingulate cortex (Milham, Banich, Claus, & Cohen, 2003; Silton, Heller, Towers, Engels, Spielberg, Edgar, … & Miller, 2010). Such a theoretical account can potentially reconcile the absence of effects of tDCS applied over the left DLPFC on Stroop performance in our and previous studies with correlational findings of neuroimaging studies showing DLPFC activity to be involved in Stroop interference (Milham et al., 2003a, b).

Interestingly, in our Stroop sequence task, reduced interference control should have led to relative speeding of responses in congruent trials following incongruent trials. So, under these specific task requirements, reduced interference control would have been beneficial in terms of response latencies. Because our findings do not suggest that the left DLPFC is causally involved in interference control, it would be informative for future research to test the modulation of Stroop sequence effects by cathodal tDCS applied to other brain areas, such as the right DLPFC (Brunoni & Vanderhasselt, 2014).

It is important to note that our finding indicating that the Stroop interference effect was unaffected by tDCS delivered to the left DLPFC contradicts the conclusions of Frings et al. (2018). Using a pre-post design, to compare only anodal and cathodal tDCS applied to this region, these investigators found increased error rates in incongruent (but not congruent) trials under cathodal stimulation, and no such increases in the anodal condition. However, while we employed the standard four-color version of the Stroop task, it remains to be seen whether this accounts for the discrepancy between our findings. Future studies could extend our design by including the Stroop version employed by Frings et al, and/or other tasks designed to capture interference control, such as the flanker (Eriksen & Eriksen, 1974) or go/no-go task (Lappin & Eriksen, 1966; Logan, Schachar, & Tannock, 1997). This would therefore provide converging evidence about the presence or absence of DLPFC tDCS effects on interference control.

## Limitations

Several potential limitations of our study should be acknowledged. Including a sham condition was necessary to enable separate comparison of effects of anodal vs cathodal tDCS. Nevertheless, use of sham conditions has been criticized because the subjective experience might be different than under active stimulation, and this might account for observed differences in cognitive task performance under the active and sham conditions (Frings et al., 2018). We assessed the subjective experience of the tDCS manipulation, and indeed found that anodal and cathodal tDCS were experienced as more arousing than sham stimulation. However, because cathodal and anodal tDCS did not differ in how arousing they were experienced, it seems unlikely that differences in subjective experience could account for effects of anodal tDCS, but not cathodal tDCS, compared with sham on Stroop task and *n*-back performance. Interestingly, participants in the cathodal condition (compared with anodal condition) indicated to have experienced higher concentration and less difficulty when working on the Stroop task. This pattern is contrary to the predicted behavioral effect of cathodal stimulation, potentially speaking to the idea of compensatory processes in other brain regions when left DLPFC activity is temporarily inhibited (Banich, 2009; Silton et al., 2010). Taken together, differences in subjective experience do not match, and thus unlikely account for, the behavioral patterns observed.

To assess executive functions, we used *n*-back performance to index working memory capacity, and the classical Stroop interference effect and Stroop sequence effects to index interference control. It has to be noted, however, that each of these cognitive tasks likely involves several distinct facets of executive functions. For instance, *n*-back performance requires a memory span depending on the level of *n*, updating information with each successive consonant presented and mentally comparing pieces of information (Oberauer, Süß, Schulze, Wilhelm, & Wittmann, 2000). Stroop performance likely relies on abilities to shield attention against distractors, and inhibiting prepotent responses (Friedman & Miyake, 2004) as well as maintaining task-related information active in working memory while competitively suppressing irrelevant information (Munakata et al., 2011) and flexibly up- and down-regulate this interference control from trial to trial (Egner, 2007). The left DLPFC might be involved only in some of these processes (Vanderhasselt et al., 2009). As such, future research may usefully incorporate tasks capturing different aspects of executive control to identify those that are and are not enhanced via tDCS. In particular, for a more complete understanding of a potential involvement of the DLPFC in inhibitory control, it is desirable to apply a wider range of tasks covering different facets of the construct (Friedmann & Miyake, 2004).

Finally, because we delivered the tDCS manipulation only to the left DLPFC, our findings do not permit conclusions concerning the role of the right DLPFC (Brunoni & Vanderhasselt, 2014). While our results do not support involvement of the left DLPFC in interference control, it remains unknown whether the right DLPFC may make a causal contribution to interference control, as Vanderhasselt et al. (2009) have argued (see also Cieslik et al., 2015). If the right DLPFC is related “to context-driven regulation and the executive modification of cognitive control” (Vanderhasselt et al., 2009), then anodal tDCS applied over that region might serve to attenuate Stroop effects indexing interference control. Hence, it would be interesting and informative to contrast anodal and cathodal stimulation of the right DLPFC with a sham condition, and test the modulation of the Stroop interference effect and Stroop sequence effects. Such a study could potentially reveal inhibitory effects of cathodal stimulation, not observed in the present study or in prior research.

## Conclusions

Our study provides evidence that anodal tDCS applied over the left DLPFC, compared with a sham procedure, served to enhance accuracy in an *n*-back task 15 minutes after active tDCS stimulation had ended. Moreover, concurrent anodal tDCS led to speeded colour classification responses in a Stroop task but did not modulate the Stroop interference effect (reflecting poorer performance on incongruent trials compared to congruent trials). Cathodal tDCS did not compromise executive functioning, as indexed by either the speed or accuracy of the cognitive tasks employed in this study. The design of this study overcomes prior methodological problems (Medina & Cason, 2016), by using a larger sample and by employing strategic placement of the reference electrode so as not to confound left DLPFC stimulation with right DLPFC inhibition. Together, the pattern of our findings suggests that the left DLPFC is causally implicated in the determination of working memory capacity but not in interference control.

## Footnotes

1 In the sham condition, participants receive a short stimulation (30 s) with fade-in and fade-out (10s each) to keep them blind to the condition they are in.

2 Similar setups were employed in studies that investigated aftereffects immediately after stimulation ended (Mulquiney et al., 2011) and in studies on immediate aftereffects using different working memory tasks than n-back (e.g., Sternberg task). There are few notable exceptions (Andrews et al., 2011; Jeon & Han, 2012; Keeser, 2011) with studies in which no tasks were administered during stimulation. Yet, in these studies, either different tasks than n-back were used to assess working memory, or aftereffects were assessed immediately after stimulation ended, thus limiting comparability of findings.

3 Due to a technical error, for some participants there were 49 congruent trials, and instead only 47 control or incongruent trials. This was taken into account when calculating accuracy and response latencies.
